# Attention Reduces Stimulus-Driven Gamma Frequency Oscillations and Spike Field Coherence in V1

**DOI:** 10.1016/j.neuron.2010.03.013

**Published:** 2010-04-15

**Authors:** Matthew Chalk, Jose L. Herrero, Mark A. Gieselmann, Louise S. Delicato, Sascha Gotthardt, Alexander Thiele

**Affiliations:** 1 Institute of Neuroscience, Newcastle University, Newcastle upon Tyne, NE2 4HH, UK; 2 School of Informatics, University of Edinburgh, Edinburgh, EH8 9AB, UK

**Keywords:** SYSNEURO

## Abstract

Rhythmic activity of neuronal ensembles has been proposed to play an important role in cognitive functions such as attention, perception, and memory. Here we investigate whether rhythmic activity in V1 of the macaque monkey (*macaca mulatta*) is affected by top-down visual attention. We measured the local field potential (LFP) and V1 spiking activity while monkeys performed an attention-demanding detection task. We show that gamma oscillations were strongly modulated by the stimulus and by attention. Stimuli that engaged inhibitory mechanisms induced the largest gamma LFP oscillations and the largest spike field coherence. Directing attention toward a visual stimulus at the receptive field of the recorded neurons decreased LFP gamma power and gamma spike field coherence. This decrease could reflect an attention-mediated reduction of surround inhibition. Changes in synchrony in V1 would thus be a byproduct of reduced inhibitory drive, rather than a mechanism that directly aids perceptual processing.

## Highlights

► Gamma synchronization in V1 depends on activation of normalization mechanisms ► Attention reduces strength of LFP gamma synchronization in V1 ► Attention reduces spike field coherence in V1 ► Increased gamma spike field coherence in is not a universal mechanism of attention

## Introduction

Attention plays an important role in conscious perception (Chun and Marois, 2002; Rensink, 2000). It ensures that sensory processing is biased toward behaviorally relevant features and locations. As a consequence the perceptual quality of a visual stimulus located within an attended region is improved (Carrasco et al., 2004) at the expense of the perceptual quality of stimuli located elsewhere (Pestilli and Carrasco, 2005). Such attention-dependent perceptual changes are reflected in neurophysiological data from striate and extrastriate visual cortex, where firing rates of neurons tuned toward an attended spatial location (Spitzer et al., 1988; Treue and Maunsell, 1996) or feature dimension (Martinez-Trujillo and Treue, 2004; McAdams and Maunsell, 2000) are increased. In addition, spatial and feature-guided attention increases the synchrony of neuronal populations in area V4 (Bichot et al., 2005; Fries et al., 2001), thereby likely increasing the impact of their action potentials at target locations. Thus, in addition to modulating neuronal firing rates, attention acts to synchronize the activity of neurons that respond to an attended stimulus (Börgers et al., 2008; Buehlmann and Deco, 2008; Buia and Tiesinga, 2006; Deco and Thiele, 2009; Fries et al., 2001; Gregoriou et al., 2009; Steinmetz et al., 2000).

These attention-dependent changes in neural synchrony have so far been recorded at mid- and high-level stages in the visual hierarchy (Bichot et al., 2005; Buschman and Miller, 2007; Fries et al., 2001; Gregoriou et al., 2009). It is unclear whether attention modulates the neural response in a similar way in early cortical processing. To test this we measured the local field potential (LFP) and spiking activity in primary visual cortex while macaque monkeys performed a visual attention-demanding task. We found that attending to a visual stimulus located within the receptive field (RF) of the recorded neurons resulted in a significant decrease in the magnitude of LFP oscillations and the spike field coherence (SFC) in the gamma frequency range (30–50 Hz). To ensure that this result was not related to task differences, we confirmed that attention increases gamma LFP power and SFC in area V4, in line with previous results (Fries et al., 2001). Thus, increases in neuronal synchrony are not a universal mechanism by which attention benefits the processing of visual stimuli in visual cortex.

## Results

We measured the LFP signal from 258 recording sites in area V1 from three macaque monkeys (monkeys HU, HO, and BL) while they engaged in a top-down spatial attention task where bar stimuli of optimal orientations were presented centered on the RF of neurons at the recording site. The attentional state of the monkey was manipulated by presenting a visual cue prior to each trial, which instructed the monkey to attend either toward or away from the corresponding RF (“attend-RF” and “attend-away” conditions, respectively). In order to investigate the impact of interaction of attention and stimulus dimension on the LFP response and the SFC, we varied the stimulus contrast in experiment 1 and the bar length in experiment 2. For experiment 1 the contrast of the bar stimuli was variable (5%, 10%, 15%, 20%, 25%, and 50% Michelson contrast), while the bar length was fixed (0.4° long in monkey HU, 39 recording sites; 1.2° long in monkey HO, 26 recording sites). In experiment 2 the length of the bar stimuli was varied (0.2°–2.4°) and the contrast was fixed at 24% in monkey HU (135 recording sites) and monkey BL (12 recording sites), while it was 85% in monkey HO (46 recording sites). For additional task and experimental details see Experimental Procedures and Figure S1.

**Figure 1:**
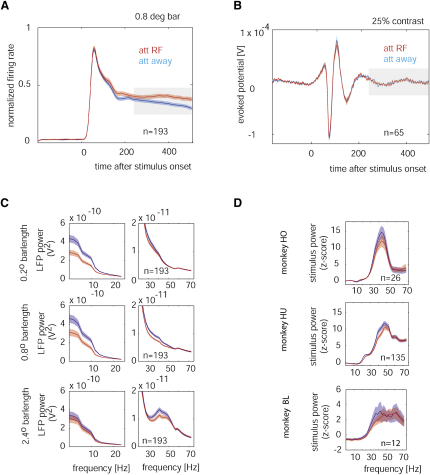
Average Spike and LFP Responses (A) Normalized multiunit signals from the bar length (0.8° long bar) experiments averaged over 193 recording sites. Red curve shows the activity in the attend-RF condition; blue curve, the activity in the attend-away condition. Red and blue shaded areas show SEM. The gray shaded area shows the time period that was used for the spectral analysis. (B) Population-evoked potential of the LFP from the contrast experiment (25% contrast). (C) Spectrum during the period from 256 to 512 ms after stimulus presentation for the attend-away (blue) and attend-RF (red) conditions from the bar length experiment. Left graphs show data from 0 to 25 Hz; right graphs, data from 20 to 72 Hz. (D) Power spectrum normalized for stimulus-induced effects, i.e., normalized by the power spectrum prior to stimulus presentation for the attend-away (blue) and attend-RF (red) conditions separately for the three monkeys. Data from monkey HO shows an example from the contrast experiment (50% stimulus contrast). Data from monkey HU and BL are from the bar length experiment (2.4° bar length). Shaded areas show SEM.

In line with the general literature, we found that attending toward the RF location increased the firing rate of the neurons compared with the rate achieved in the attend-away condition. An example of this effect in the recorded population (bar length experiment) is shown in Figure 1A (p < 0.001, signed rank test). Figure 1B shows an example, population-evoked LFP response from the contrast experiment. It shows that stimulus onset resulted in a stereotypical deflection of the LFP, which lasted for about 200–250 ms, whereas after the LFP, as assessed by the evoked response, it was reasonably stationary. The latter is a prerequisite for performing the spectral analyses that are reported below. To investigate the effects of attention on the sustained LFP response, we calculated the LFP response power spectrum and the stimulus-induced power spectrum, both averaged over the time interval of 256–512 ms after stimulus presentation. The time period of 256–512 ms was chosen because it is the period wherein attentional modulation of firing rates was most profound (Herrero et al., 2008; Roberts et al., 2007) (see also Figure 1A), and it corresponds to the time period wherein the animal had to increase the level of attention to detect behaviorally relevant stimulus changes (Roberts et al., 2007). Figure 1C shows the average LFP power spectrum for the recordings from the bar length experiment, pooled across the three monkeys. The attend-away condition is shown in blue, and the attend-RF condition is shown in red. Power spectra exhibited their maximum at low frequency, dropping off with increasing frequency. However, there was an additional peak in the gamma band (30–50 Hz), provided that the stimulus induced a V1 network state that favored gamma oscillations (Gieselmann and Thiele, 2008), i.e., a large stimulus was used that encroached on the suppressive surround. In addition to the dependence on stimulus type, gamma oscillations were usually larger for the attend-away, compared with the attend-RF, condition. These differences were significant (p < 0.05, two-factor repeated-measurement [RM] ANOVA; for a detailed analysis of the significance levels for different spectral bands, see below). Figure 1D shows examples of stimulus-induced spectra for the three animals. The attend-away condition resulted in more stimulus-induced power in the gamma range than the attend-RF condition.

In order to provide a quantitative understanding of how attention modulated the LFP signal, we divided the power spectrum into five different frequency bands (delta: 1–4 Hz, theta (cortical): 4–7 Hz, alpha: 7–13 Hz, beta: 13–25 Hz, and gamma: 30–50 Hz), and analyzed the effects of attention on the LFP response power separately for each frequency band. The gamma range was restricted to powers of <50 Hz, because all three monkeys showed that their main stimulus-induced gamma activity within this range (see Figure 1D). Moreover, the LFP power for higher frequencies could be contaminated by “spike intrusion,” and would thus at least partially reflect the multiunit activity at the recording site.

**Figure 2:**
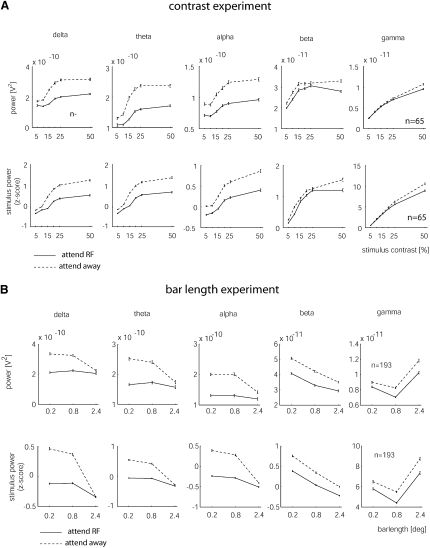
Influence of Stimulus and Attention on Spectral LFP Power (A) Effects of attention and stimulus contrast on different frequency bands of the raw and stimulus-induced LFP power for the contrast experiment. The population raw LFPs of the delta, theta, alpha, beta, and gamma powers as a function of contrast is shown in the upper plots. The stimulus-induced power for these frequencies as a function of stimulus contrast is shown in the lower plots. (B) Effects of attention and stimulus length on different frequency bands of the raw and stimulus-induced LFP power for the bar length experiment. The population raw LFPs of the delta, theta, alpha, beta, and gamma powers as a function of stimulus length is shown in the upper plots. The stimulus-induced power for these frequencies as a function of stimulus length is shown in the lower plots. Data for the attend-RF condition are shown in solid black, whereas data for the attend-away condition are shown in dashed black. Error bars show SEM.

Data from the experiment wherein stimulus contrast was systematically varied are shown in Figure 2A. Stimulus contrast significantly increased delta, theta, alpha, beta, and gamma band activity, while attending to the RF significantly reduced power in all five frequency bands (two-factor RM ANOVA; delta: p_attention_ < 0.001, p_contrast_ < 0.001, theta: p_attention_ < 0.001, p_contrast_ < 0.001, alpha: p_attention_ < 0.001, p_contrast_ < 0.001, beta: p_attention_ < 0.001, p_contrast_ < 0.001, gamma: p_attention_ = 0.004, p_contrast_ < 0.001, n = 65 recording sites). The effects for the gamma band activity were significant both collectively and individually (for additional details see Table 1), and they applied to both the raw power and the stimulus-induced power. In addition to the effects of contrast and attention alone, we also found a significant interaction between stimulus contrast and attention for the gamma band activity. Attention reduced the raw and stimulus-induced power more strongly at high contrasts (raw gamma power: p_attention^∗^contrast_ = 0.001; stimulus-induced gamma power: p_attention^∗^contrast_ < 0.001).

The data for the experiment wherein the bar length was varied are shown in Figure 2B. Bar length had a significant effect on all frequency bands (p < 0.001, two-factor RM ANOVA, n = 193 recording sites from three monkeys). However, increasing stimulus size did not have the same effect on the delta, theta, alpha, and beta band activity as increasing stimulus contrast did. While increasing contrast resulted in more delta, theta, alpha, and beta LFP power, increasing the stimulus size significantly reduced the overall power and the stimulus-induced power in the delta, theta, alpha, and beta bands (p < 0.001, two-factor RM ANOVA). Notably, the effects of contrast and stimulus length on gamma band activity were very similar. LFP power significantly increased with bar length in the gamma band (p < 0.001, two-factor RM ANOVA). In line with the contrast experiments, attending to the RF of the recording sites significantly reduced the power in the delta, theta, alpha, beta, and gamma bands (p < 0.001, two-factor RM ANOVA) in monkey HU and monkey BL. Additionally, a significant interaction between bar length and attention was found for all five frequency bands (p < 0.05, two-factor RM ANOVA). In the delta, theta, alpha, and beta band, the largest attention-induced power changes occurred for short bar stimuli, while in the gamma band, the largest changes occurred for long bars. The effects in monkey HO were more variable (Table 1; see Figure S2 for a possible explanation of this variability).

The stimulus-induced power is dependent on the power during spontaneous activity (see Experimental Procedures). It is conceivable that attending to the cued location already caused an increase in gamma power before stimulus onset, and thus stimulus-induced power reduction might be a consequence of increased gamma power before stimulus onset. However, we found no significant increase of gamma power before stimulus onset for the two attention conditions (p > 0.4, Wilcoxon signed rank test). Thus, the change in stimulus-induced power was not a result of changes occurring prior to stimulus onset. Another way of controlling for this is by calculating stimulus-induced power with explicit normalization relative to power during spontaneous activity (see Experimental Procedures). An approach where stimulus power was calculated with explicit normalization relative to power during spontaneous activity yielded basically identical results to an approach where this explicit normalization was not performed (for details see Experimental Procedures).

### Influence of Stimulus Type and Attention on SFC

**Figure 3:**
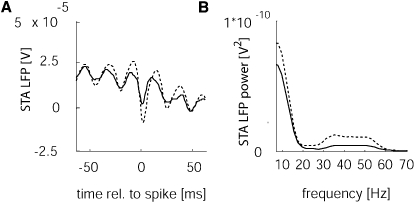
Effect of Attention on the Spike Triggered Average (STA) LFP for an Example Recording Site (Monkey HU, 2.4° Bar Length) (A) Attention reduced the STA LFP response (compare solid [attend-RF condition] versus dashed [attend-away condition]). (B) Power spectrum calculated from the STA LFP response in (A). Attention strongly reduced the STA LFP power across most frequencies shown; this was most pronounced in the alpha and the gamma frequency bands.

The previous analyses have determined the power spectrum of the LFP to be a function of the stimulus and of attention. The influence of attention on oscillatory behavior in area V4 has previously been analyzed by calculating the power spectrum of the spike triggered average (STA) LFP and by calculating the SFC (Fries et al., 2001). This study had found that the SFC was increased in the gamma range in V4 when attention was directed to the RF of the neurons under study. Although it is unlikely that LFP gamma power decreases with attention in V1 while SFC in the gamma range shows a concomitant increase, we still aimed to determine the effect of stimulus type and attention on SFC. Figure 3A shows an example of the spike triggered LFP from a single recording site, when a bar of 2.4° was presented and attention was either directed to the RF of the recording site (solid) or directed away from the recording site (dashed). Figure 3B shows the power spectrum that was obtained from the STA at this recording site. The STA LFP in the attend-away condition was more strongly modulated in the gamma frequency range than in the attend-RF condition, and consequently the power spectrum showed a larger peak in the gamma range for the attend-away condition (Figure 3B).

**Figure 4:**
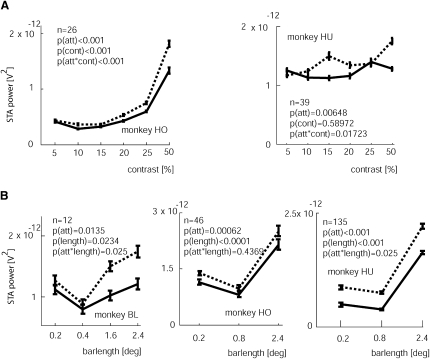
Power of the STA LFP in the Gamma Range for the Different Experiments (A) Stimulus contrast varied. (B) Bar length varied. Data are shown separately for the different monkeys as a function of attention (solid lines: attend-RF; dashed lines: attend-away). For both experiments and all monkeys, attention significantly reduced the STA LFP power in the gamma range. Additionally, a significant interaction between attention and stimulus type occurred for most conditions. p values (two-factor RM ANOVA) denote whether attention or stimulus type had a significant influence on STA LFP gamma power or whether there was an interaction between attention and stimulus type. n denotes the number of recording sites contributing to the sample. Error bars show SEM.

Figure 4 shows the effect of stimulus type and attention on the power in the gamma range of the STA LFP for all recording sites separately for the different monkeys. Attention significantly reduced the STA gamma power for the experiments wherein stimulus contrast was varied in both monkeys (p < 0.01, two-factor RM ANOVA). Moreover, for these experiments there was a significant interaction between contrast and attention in both animals (p < 0.05, two-factor RM ANOVA). The differences between the data from the two monkeys that are apparent from Figure 4A are probably due to the fact that the stimulus size differed. In monkey HO a bar of 1.2° length was used, which activates center surround modulation, provided the contrast is high. In monkey HU a bar of 0.4° length was used, which hardly (if at all) activates center surround mechanisms, and therefore does not generate as much gamma drive per se. Therefore increasing contrast in monkey HU does not result in the same STA gamma power increase as in monkey HO. However, irrespective of the stimulus-induced difference, attending to the RF resulted in significantly reduced STA LFP gamma power in both animals.

Attention significantly reduced STA LFP gamma power in all three monkeys when the bar length was varied (p < 0.05, two-factor RM ANOVA, Figure 4B). Stimulus type also had a significant effect on STA LFP gamma power. Increasing the bar length to a size where the ends just encroached on the RF surrounds (0.4° or 0.8° long) initially reduced STA LFP gamma power, while large bars (which extended well into the RF surrounds of V1 neurons) resulted in significantly increased STA LFP gamma power (Figure 4B).

**Figure 5:**
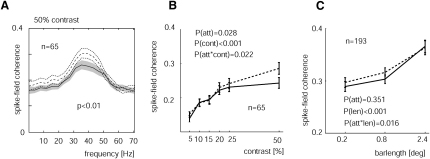
SFC as a Function of Attention (A) SFC for a 50% contrast stimulus averaged across all recording sites from monkey HU and monkey HO. SFC was strongest in the gamma range (30–50 Hz, gray bar at the bottom of the graph). Attending to the RF resulted in lower SFC (solid lines, gray shaded area) compared with that resulting from attending away (dashed lines). Shaded (dashed) area shows SEM. (B) Average SFC in the gamma range as a function of stimulus contrast and attention. (C) Average SFC in the gamma range as a function of stimulus size and attention. Solid lines show attend-RF conditions; dashed lines, attend-away conditions. p values (two-factor RM ANOVA) denote whether attention or stimulus type had a significant influence on SFC in the gamma range or whether there was an interaction between attention and stimulus type. n denotes the number of recording sites contributing to the sample. Error bars show SEM.

In addition to the STA LFP gamma power, we also calculated the SFC (see Experimental Procedures). Figure 5A shows the SFC for the attend-RF (solid line) and attend-away (dashed line) condition when a bar of 50% luminance contrast was presented, pooled across data from both monkeys. SFC showed a peak in the gamma range (∼30–50 Hz), and this was significantly larger for the attend-away condition (p < 0.01, Wilcoxon signed rank test). The SFC data in the gamma range as a function of contrast for the two monkeys are shown in Figure 5B. SFC significantly increased as stimulus contrast increased (p < 0.001, two-factor RM ANOVA). Attending to the RF significantly decreased SFCs (p = 0.03, two-factor RM ANOVA), and there was a significant interaction between contrast and attention (p = 0.02, two-factor RM ANOVA).

When stimulus length was varied, we equally found a significant effect of stimulus length on the strength of the SFC (Figure 5C). Larger stimuli resulted in significantly higher SFC (p < 0.0001, two-factor RM ANOVA). Attention only decreased SFC for shorter bar lengths. Attention itself had no significant effect on SFCs in the gamma range, but there was a significant interaction between attention and bar length (p = 0.016, two-factor RM ANOVA), reflecting a stronger effect of attention on SFC for short and medium-sized bars (see Table 1 for a breakdown of effects across different animals).

**Figure 6:**
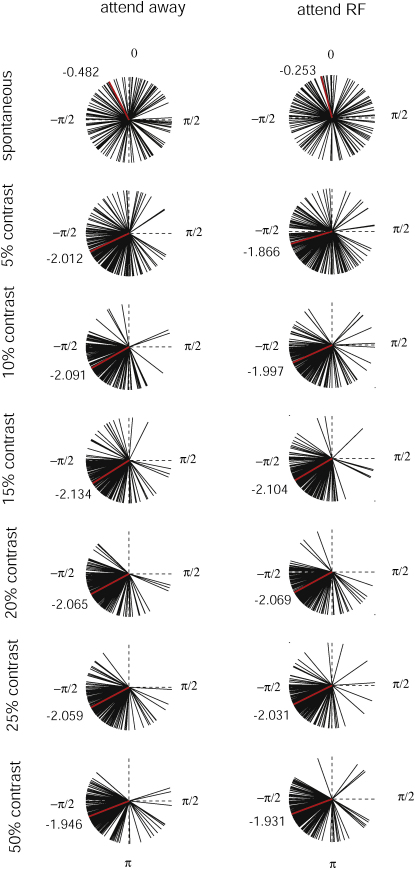
Spike-Field Phase Relationship for the Gamma Range as a Function of Contrast and Attention Each recording site contributes three vectors, obtained from three different parts of the gamma frequency band (36–40, 40–44, and 44–48 Hz). The reason for this is that the phase angle from the three frequency bands cannot be averaged (a random phase angle would average to 0). Columns show different attention conditions; rows, different stimulus conditions. During spontaneous activity no specific angle for the spike-field phase relationship was apparent. Upon stimulus presentation spike-field phase relationships concentrated at ∼−0.65π. Attention had no significant influence on the median spike-field phase relationship. The red vector and number insets give the median spike-field phase relationship.

While these data show that attending away from the RF increases LFP gamma power and SFC in V1, it may also be important to look at the spike-field phase relationship. It has been argued that gamma power ensures that spikes are elicited at a “good” LFP phase, so that neuronal interactions are more effective. It may be the case that despite increased SFC in the attend-away condition, the phase relationship is more effective to promote neuronal interactions in the attend-RF condition. While it is unclear what an ‘optimal’ phase relationship is, as this will depend on conduction delay times, it is still possible to investigate whether attention affects the phase relationship between spikes and gamma frequency oscillations. We thus calculated the spike-field phase relationship for the attend-RF and attend-away condition for each experiment as a function of stimulus condition. Figure 6 plots the distribution of spike-field phase relationship for the contrast experiment. For this figure we plotted the preferred phase relationship for the frequency range from 36–40 Hz, from 40–44 Hz, and from 44–48 Hz, i.e., each recording site contributes three data points (vectors) for each stimulus and attention condition. A plot where the individual frequency bands are analyzed separately yields virtual identical results. The stimulus type had a significant effect on the spike-field relationship (p < 0.001, two-factor ANOVA, contrast experiment; p = 0.02, two-factor ANOVA, bar length experiment). During spontaneous activity there is no consistent spike-field phase relationship. Upon stimulus presentation there is a concentration of spike-field phase relationships at an angle of ∼−0.65π. Upon inspection of Figure 6 it is also clear that the concentration is more profound for medium and high contrast stimuli. Attention had no significant effect on the distribution of spike-field phase relationships (p = 0.26, two-factor ANOVA). Also, there was no significant interaction between stimulus condition and attention on spike-field phase relationships (p = 0.87, two-factor ANOVA). The results from the bar length experiment yielded virtually identical results. We thus conclude that attention reduces SFC in V1 but does not systematically affect the spike-field phase relationship.

### Attention and LFP Gamma Power in Area V4

**Figure 7:**
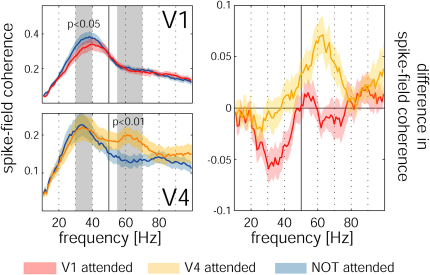
SFC of Simultaneous Recordings in V1 and V4 (n = 48) SFC in V1 was significantly reduced with attention in the frequency range from 30–40 Hz (p < 0.05, signed rank test), but it was significantly increased with attention in V4 in the frequency band of 55–70 Hz. The left side shows z-transformed average SFC for the two areas. The right graph shows the attention-induced difference in SFC for the two areas. Shaded areas show SEM.

To ensure that the results obtained in V1 were not caused by differences in experimental design or laboratory differences, we recorded LFPs from an additional monkey (monkey ST) in an attention task in V4 at 43 recording sites. Stimuli were moving square-wave gratings within a circular aperture of 2° diameter (1 cyc/°, 2 Hz). As in the task used for the V1 recordings, the monkey was cued to attend to either the RF at the recording site or a location in the opposite hemifield. He had to detect a reduction in grating contrast from 90% to 60% Michelson contrast. For these recordings we found a significant increase in LFP gamma power when the animal directed attention to the RF of the recording sites, compared with when he attended away from it (p < 0.001, Wilcoxon signed rank test). However, it could be argued that the animal differed from those used for the V1 study and the stimuli were also different. Thus task difficulty and requirements may also have differed, and this might be the cause of the different results in V1 and V4. To determine whether this can account for the different results, we recorded from monkey HU's area V1 and area V4 simultaneously, under task conditions that were identical to those described for the previous V1 experiments, but under stimulus conditions that were slightly different. Instead of using small bars, we used square-wave gratings presented in a circular aperture of 1° diameter. The animal had to detect a subtle change at the center of the cued grating and ignore changes at the other grating location. We used slightly more extended stimuli to ensure that simultaneously recorded neurons in V1 and V4 would both be activated. The RFs of neurons in V1 and V4 in all these recordings were overlapping, although RF centers did not necessarily coincide. Stimuli were always centered on the RF of the V1 neurons, but always also elicited a significant response in the simultaneously recorded V4 neurons. These simultaneous recordings replicated our basic V1 results, namely that attention to the RF of the neurons under study reduced SFC (p < 0.05, signed rank test, n = 48 recording sites), while in V4 the simultaneously recorded SFC was significantly increased (p < 0.01, signed rank test, n = 48 recording sites). The results are shown in Figure 7. From Figure 7 it is also apparent that while attention affected SFC differently in V1 and V4, the effects occurred at different frequencies. In V1 the main effect occurred at a frequency of 30–40 Hz in monkey HU, while in V4 it occurred at a frequency of 55–70 Hz. Since these data come from simultaneous recordings in monkey HU, it cannot be argued that stimulus, task, behavioral, or individual differences contributed to the differences seen in V1 versus V4. Thus, attention reduces SFC in V1, which differs from previous (and our own) findings in V4.

## Discussion

We found that raw and stimulus-induced gamma power, as well as the STA LFP gamma power and the SFC in V1, increased with bar length and with stimulus contrast. The largest increases occurred for high contrasts invoking contrast normalization mechanisms, or long bars exceeding the classical RF. Attention decreased the gamma band power as well as SFC in the gamma range, whereas it had no effect on the spike-field phase relationship per se. Generally the largest attention-induced decreases of gamma power occurred for long bars and high contrast.

Our data relating to the dependence of gamma power on stimulus types are in line with results of two recent studies (Gieselmann and Thiele, 2008; Henrie and Shapley, 2005). Gieselmann and Thiele (2008) found that the magnitude of gamma frequency LFP activity increased monotonically for all stimulus sizes, with maximal increases occurring for stimuli that infringed on the classical RF surround, where suppression begins to dominate the spiking activity. Henrie and Shapley (2005) found that LFP gamma oscillations increased monotonically with stimulus contrast. Maximal increases occurred at high stimulus contrasts when the single-unit activity saturated and where contrast normalization mechanisms (Heeger, 1992; Henrie and Shapley, 2005; Sceniak et al., 2001; Thiele et al., 2004) with recurrent inhibitory activity begin to dominate. In agreement with modeling and in vitro studies (Tiesinga and Sejnowski, 2004; Traub et al., 1996; Whittington and Traub, 2003), this suggests that the magnitude of the LFP response in the gamma frequency range is determined by the summed contributions of excitatory and inhibitory activity from both the classical and extraclassical RF, with recurrent inhibitory activity from the extraclassical RF playing a dominant role. At first glance this contradicts the notion that strong stimuli result in reduced magnitude and spatial extent of lateral interactions in V1 (Nauhaus et al., 2009). Nauhaus et al. (2009) found maximal *facilitatory* interactions at low contrast, and reduced interactions at high contrast. One suggested possibility for this result was recruitment of a disynaptic inhibitory signal at high contrast. Such inhibitory recruitment could drive gamma oscillations. However, both results (increased gamma oscillations and reduced lateral interactions) could also arise within an inhibition-stabilized network (Ozeki et al., 2009). Here surround suppression briefly increases the overall inhibitory drive, which then quickly causes overall reduction of excitation and inhibition within the network (Ozeki et al., 2009). Reduced excitation and inhibition with high contrast stimuli would yield smaller space constants and a smaller magnitude of lateral interactions (Nauhaus et al., 2009). Because reduced inhibition can also be a prerequisite for gamma oscillations (Börgers et al., 2008), an inhibition-stabilized network could favor gamma oscillations when surround suppression or contrast normalization mechanisms are activated.

Our data relating the dependence of gamma power on attention are at odds with data from extrastriate cortex (Fries et al., 2001). Data from V4 demonstrated an increase in SFC in the gamma range when attention was directed to the RF of the neurons under study (Fries et al., 2001). It has been suggested that increased SFC in the gamma range would promote an increased impact of excitatory postsynaptic potentials at target neurons, and thus improve neuronal communication (Börgers et al., 2008; Womelsdorf and Fries, 2007). Contrary to these findings, our data from V1 showed decreased LFP gamma power and decreased SFC in the gamma range when attention was directed to the RF of the recorded neurons. This discrepancy cannot be explained by experimental approaches (or laboratory differences) because we found increased LFP gamma band activity in V4 when attention was directed toward the RF of the recorded neurons, while simultaneously recorded V1 data showed the opposite result.

Despite the decrease of gamma power with attention in V1, we still found an increase in neuronal firing rate with attention. Thus, attention increases the firing rate of neurons representing the attended stimulus while simultaneously decreasing the LFP gamma power and the SFC in the gamma range in V1. What could be the mechanisms behind this dissociation and behind the difference between the V1 and V4 results? We will first speculate on possible mechanisms that might promote increased firing rates when gamma oscillations are decreased, followed by possible reasons for V1 versus V4 differences.

We have recently shown that increases in LFP gamma oscillations are paralleled by decreased firing rates in V1 (Gieselmann and Thiele, 2008). Both changes are likely due to recruitment of inhibitory interneurons from neighboring hypercolumns when the neuron's suppressive surround is stimulated. This inhibition causes reduced firing rates, but can simultaneously strengthen pyramidal-interneuron gamma (PING) oscillations (Börgers and Kopell, 2005; Gieselmann and Thiele, 2008). If attention reduced the surround suppression, it could increase firing rates and decrease gamma oscillations simultaneously, as found in our data. It has recently been shown that attention affects center surround mechanisms in primary visual cortex (Roberts et al., 2007) and in V4 (Sundberg et al., 2009). In area V1 attention affected center surround integration by reducing neurons' summation area (at parafoveal sites), not by reducing surround suppression (Roberts et al., 2007). The results presented here suggest that attention can also reduce inhibitory surround mechanisms in primary visual cortex. As a consequence, gamma oscillations would be diminished. At first glance this scenario is incompatible with recent modeling work arguing that a release from inhibition causes increased, not decreased, gamma oscillations in extrastriate areas (Börgers et al., 2008). However, the modeling proposes a very local mechanism for the increased gamma oscillations, probably restricted to the representation of the classical RF of the recorded neurons. Within that modeling framework attention reduces the drive to local inhibitory interneurons, releasing pyramidal cells from a bath of inhibition (Börgers et al., 2008). Such a release could be mediated through muscarinic mechanisms (Börgers et al., 2008; Xiang et al., 1998, 2002), which contribute to attention in V1 (Herrero et al., 2008). Thus, attention could in theory reduce surround suppression, causing reduced gamma oscillations at a larger scale, and simultaneously increase gamma oscillations at a very local network level. Because the recorded LFP is widely assumed to be the sum of activity from ∼0.5–1.2 mm of cortical tissue surrounding the electrode (Berens et al., 2008; Gieselmann and Thiele, 2008), a possible increase in very local gamma may be concealed by a more global decrease of gamma oscillations. Although we cannot entirely exclude this possibility, we also found decreased gamma LFP power and SFC with attention for stimuli that were entirely restricted to the classical RF of the recorded neurons (0.2°–0.4° bar length; see, e.g., Figures 4 and 5). The latter makes a scenario of very local increases of gamma oscillations unlikely.

Rather than affecting inhibitory drive, attention could increase “stimulus-driven” feedforward activity relative to “expectation-driven” recurrent or top-down feedback (Sarter et al., 2005; Yu and Dayan, 2005). If true, we would expect an increase in feedforward excitatory activity and possibly reduced recurrent inhibitory activity with attention. This could then decrease the level of oscillations within the network (Traub et al., 1996). Whether any of these proposed events capture the underlying mechanisms of the reduced gamma oscillations with attention in V1 remains to be determined in future experiments.

Our above proposal, that attention reduced inhibitory drive, seems at odds with recent models of attention. Normalization models of attention (Lee and Maunsell, 2009; Reynolds and Heeger, 2009) assume that attention increases normalization, i.e., increases the inhibitory drive. This should increase gamma oscillation, rather than decrease it. Thus, normalization models may be able to explain changes in gamma oscillations in area V4, but they are not a straightforward model to explain our V1 results. However, at the same time, the increased inhibition could affect surrounding cortical locations, and suppress local activity at those locations. Reciprocal suppression from the surrounding cortical locations to the attended location would then be reduced, and thus the overall inhibitory drive in the network would be smaller, resulting in reduced gamma power. The gamma power reduction would then be a byproduct of the reduction of surround influences. The input gain model of spatial attention is similar in flavor (Ghose, 2009). It assumes that attention increases the strength of excitatory and inhibitory inputs at the attended location. Depending on how the model is interpreted, it could also yield a scenario wherein reciprocal inhibition between neurons at the attended location and locations in the surround is reduced, which could then result in reduced gamma oscillations with attention. Thus, depending on how the attention models are interpreted, they may be able to account for the results presented here. However, it should be kept in mind that these models were not developed to account for change in neuronal synchrony with attention, but rather for firing rate changes under a variety of different stimulus and task conditions. To mechanistically account for the data reported here, it will be necessary to develop models that explicitly investigate oscillatory behavior of neuronal networks.

Our finding that attention decreases the degree of neuronal synchrony in V1 in the gamma range conflicts with results from higher areas within the visual processing hierarchy (Buschman and Miller, 2007; Fries et al., 2001; Gregoriou et al., 2009), demonstrating that the effects of attention on the synchrony of network activity are heterogeneous within visual cortex. As argued above, we currently favor the interpretation that attention in V1 results in reduced center surround inhibition, with a consequence of reduced gamma oscillations, provided an experimental design is used wherein attention is tightly focused at the center of the classical RF. As shown by others (Fries et al., 2001) (and also evident from our own V4 data), attention in V4 increases gamma oscillations. Thus, either attention may have different effects on center surround mechanisms in V1 and in V4, or the respective center surround structures are organized differently in these areas. Yet another alternative is that inhibitory mechanisms, and thus their involvement in gamma oscillations, are differently recruited by attention in different cortical areas. This scenario is by no means unlikely, because, for example, cholinergic receptors reside on different neuronal classes and locations in macaque V1 and V2 (Disney and Aoki, 2008; Disney et al., 2006). Because cholinergic mechanisms contribute to gamma oscillations in cat visual cortex (Munk et al., 1996; Rodriguez et al., 2004), and have been proposed to contribute to attention-induced gamma oscillations (Börgers et al., 2008), differences in their local distribution could have profound implications in how they alter the local network state when recruited by attention.

Known differences in feedback from frontal and parietal cortex to V4 and V1 could also account for differences between V1 and V4. V1 does not have direct feedback from the frontal or the parietal cortex, whereas V4 has strong feedback from the frontal cortex (Felleman and Van Essen, 1991). Gregoriou et al. (2009) showed that increases in gamma power with attention in V4 are at least partly driven by feedback from the frontal eye field. However, because V1 has strong feedback from V2, MT, and V4 (Felleman and Van Essen, 1991; Hupé et al., 1998), one might assume that changes of gamma oscillations with attention in V4, MT, V2, or a combination thereof might also be fed back to V1. However, our data failed to provide evidence for this, and a more detailed understanding of the specific roles of feedback from different areas will be necessary to account for this.

Whatever the underlying mechanisms, our data from V1 are difficult to reconcile with the idea that neuronal synchronization is a universal mechanism by which behaviorally relevant signals are amplified in the cortex. It seems more likely that changes in the degree of synchrony in V1 come about as a byproduct of underlying changes in the overall dynamics of network activity due to attention. Our data suggest that attention reduces the strength of inhibitory mechanisms, and simultaneously increases excitatory drive locally (Roberts et al., 2007; Roelfsema et al., 1998).

The finding that attention increases the degree of synchrony in V4 has been used as support for the hypothesis that attention provides the top-down signal required to implement perceptual “binding by synchrony.” Attention would thereby act to selectively synchronize the activity of neurons that respond to different aspects of an attended stimulus, so that the activity of these neurons is “bound” together and can be combined appropriately during the decoding process to produce a single cognitive percept (Engel et al., 2001). However, if in V1 the degree of synchrony is reduced with attention, then this suggests that a different mechanism is required to explain how perceptual binding is implemented in the earliest visual areas, and it implies that neuronal synchrony is not a general mechanism by which this is achieved.

In summary, we found that directing attention toward the RF of neurons located adjacent to the recording electrode significantly reduced the gamma LFP response and the SFC. This attention-dependent modulation of the gamma LFP power increased with stimulus size and stimulus contrast, and for the SFC, it increased with contrast, but was more profound for stimuli confined to the RF. Moreover, the LFP and SFC gamma band response increased monotonically with bar length and stimulus contrast, suggesting that it represented the activity of excitatory and inhibitory neurons summed over a region that included both the classical and extraclassical RF. Because directing attention toward the RF of V1 neurons increases their firing rate, the attention-dependent decrease in low gamma band LFP oscillations was due to a decrease in the synchronization of spiking activity within the network, rather than an overall decrease in neuronal activity. Changes in synchrony in V1 likely come about as a byproduct of other attention-dependent effects on activity within the network, such as a change in the balance between excitatory and inhibitory activity, rather than playing a direct functional role in mediating the effects of attention on perceptual processing.

## Experimental Procedures

All experiments were carried out in accordance with the European Communities Council Directive 1986 (86/609/EEC), the US National Institutes of Health Guidelines for the Care and Use of Animals for Experimental Procedures, and the UK Animals Scientific Procedures Act.

### Surgical Preparation

Monkeys (*macaca mulatta*, male, 5–8 years old) were implanted with a head holder, eye coil, and recording chambers above V1 and V4 under general anesthesia and sterile conditions. All details regarding surgical procedures, postoperative care, and the cleaning of the implant and recording chambers are published elsewhere (Thiele et al., 2006).

### Electrophysiological Recordings

We used tungsten-in-glass microelectrodes (0.5–2 MΩ, made in-house) for recording extracellular spiking activity and the LFP. Remote Cortex 5.95 (Laboratory of Neuropsychology, National Institute for Mental Health, Bethesda, MD) was used for stimulus presentation and behavioral data collection. Neuronal data were collected by Cheetah data acquisition (Neuralynx) interlinked with Remote Cortex 5.95 (Laboratory of Neuropsychology, National Institute for Mental Health, Bethesda, MD). Spike waveforms were sampled at 30 kHz. In postprocessing, spike times were sampled at 1 kHz resolution. LFP data were sampled continuously at a sampling rate of 1 kHz.

### RF Mapping

RFs of neurons surrounding the electrode tip were mapped by presenting a 0.1° black (100% contrast) square at pseudorandom locations on a 10 × 10 grid (i.e., a 1° × 1° area; five repetitions at each location; 100 ms presentation time with 100 ms gaps), while monkeys fixated centrally on the cathode ray tube (CRT). The mean response at each stimulus location (calculated from 30–100 ms after stimulus onset) was determined and a 2D Gaussian was fitted to the response distribution. The RF center was taken as the location of the peak of the fitted Gaussian.

### Main Experimental Task and Recording Protocol

For each recording site we initially mapped the RF of the extracellular action potentials from neurons in the immediate vicinity of the electrode tip, followed by determination of orientation tuning (see Gieselmann and Thiele, 2008 for details). In the main experiment the monkeys had to detect a small change in luminance at a cued (attended) location, while ignoring a change that occurred at a noncued location. Monkeys initiated trials by holding a touch bar and fixating a red fixation point (FP, 0.1° diameter) on a gray background (21 cd/m^2^) presented centrally on a 20” analog CRT monitor (110 Hz, 1600^∗^1200 pixels, 57 cm from the animal). Eye position was monitored with a camera-based system (Thomas Recording) with a fixation window of ±0.5°–0.7° in monkeys HU and HO, whereas it was monitored with a scleral search coil and a fixation window of ±0.5° in monkey BL. The animal's eye position had to remain within the fixation window boundaries throughout the trial. A cue (blue annulus, 0.24° outer diameter, 0.18° inner diameter) was presented for 400 ms on one side of the fixation spot, at a quarter of the distance to the RF center from the fixation spot (see Figure S1). The cue thus “pointed” toward the location to which the monkey had to attend. The cue was displaced either toward or away from the RF to indicate whether attention should be directed toward or away from the stimulus presented in the RF. After cue offset a 900 ms (250 ms in monkey BL) blank period occurred with just the FP present. Spatial and temporal separation of the cue from the test stimuli ensured that it had no direct effect on the neuronal response to the test stimulus. Thereafter, two identical stimuli were presented (test stimuli), one centered on the RF, the other at the same eccentricity in the opposite hemifield. Test stimuli were dark bars of preferred orientation and varying length or varying contrast (see below). After 500–800 ms (randomly assigned in steps of 1 ms), a brighter patch (0.1°^∗^0.1° wide) appeared at the center of one of the bars. The patch was always exactly at the bar center, i.e., the animal was always required to monitor the bar center, and could in principle have ignored the rest of the bar. If presented in the cued location it is referred to as “target,” and if presented in the uncued location it is referred to as “distracter.” After the presentation of a target, the monkey had to release the touch bar within 500 ms to receive a juice reward. If a distracter was presented first, the monkey had to continue to hold the touch bar and maintain fixation until target appearance. This occurred 1000–1300 ms after the distracter appeared (randomly assigned in steps of 1 ms). If the monkey made no response, the trial was terminated 500 ms after presentation of the target or distracter, whichever appeared last. Touch bar releases (correctly or incorrectly) or failure to maintain fixation resulted in immediate trial termination.

Attentional cueing was done in a blocked design. Blocks were counterbalanced in random order. Conditions of cueing toward the location of the RF are labeled attend-RF, and conditions of cueing toward the opposite hemifield are labeled attend-away. Within each block, either bar length or stimulus contrast was varied. In the length-tuning experiments, three to seven different bar lengths were used. These were chosen from 0.1°, 0.2°, 0.4°, 0.6°, 0.8°, 1.6°, and 2.4°; with a bar width of 0.1°. Three bars were used in all experiments; these were 0.2°, 0.8°, and 2.4° in monkeys HU and HO, whereas they were 0.2°, 0.4°, 1.6°, and 2.4° in monkey BL (i.e., in monkey BL four bars were used in all experiments, thus the four data points in Figure 5B). We treated the 1.6° bar data from monkey BL as if it had been from 0.8°-long bars (treating the 0.4° bar in the same manner did not change the overall conclusions). In the contrast experiment bar length was fixed (either 0.4°^∗^0.1° [monkey HU], or 1.2°^∗^0.1° [monkey HO], presented at the preferred orientation), whereas stimulus contrast was varied (5%, 10%, 15%, 20%, 25%, or 50% Michelson contrast). For each stimulus condition, the target occurred once at 500–800 ms after bar onset (early target condition) and once at 1500–2100 ms after test bar onset (late target condition). Conditions (different bar length or contrast; early or late target, respectively) were presented in pseudorandom order within each block. If the monkey made an error, the condition would be repeated later in the block. The design of the experiment ensured that an equal number of trials were obtained for all attention and stimulus conditions. Thus, the spectral data were obtained from a balanced data set and are thus unlikely to be affected. For additional details regarding the stimuli and the task, see Supplemental Information.

### Data Analysis

The LFP signal was band-pass filtered between 1–100 Hz (using a 6^th^ order Butterworth filter) to remove low-frequency direct current fluctuations and reduce high-frequency noise. Then 50 Hz power line noise was removed by applying a band pass-filter (49–51 Hz, 3^rd^ order Butterworth filter) to the original data, and subtracting the resulting filtered signal from the original data.

Spike data from the same recording electrode were obtained by band-pass filtering the raw electrode signal from 600–9000 Hz. Multiunit activity was then obtained by thresholding these filtered data, with a threshold located at ∼2 times the signal fluctuations when no background or stimulus-driven activity was present.

Because we were mainly interested in the sustained LFP response after stimulus presentation, we focused on a time window ranging from 256 to 512 ms following stimulus onset for the LFP analysis (for control purpose we also used time windows of 200–550 ms after stimulus onset and 300–500 ms after stimulus onset, both of which gave virtually identical results to the data reported in the Results section). For each trial, the raw power spectral density of the LFP response (*RPS*) over the time period of 256–512 ms after stimulus onset was estimated using a multitaper technique (Percival and Walden, 1993). For each recording site the mean power spectrum (*PS_M_*) was then calculated from the single-trial *RPS* data.

To calculate the STA LFP, we used spikes occurring within 264–460 ms after stimulus onset. We added all LFP fragments surrounding a spike occurrence by ±64 ms for all trials. This ensured that LFP data from 200–524 ms after stimulus onset contributed to our spectral power estimate. We then divided this single-trial STA LFP by the total number of spikes. The average STA LFP as a function of stimulus and attentional condition was then subjected to the same multitaper analysis described above.

For the analysis of the SFC, we binned the single trial multiunit spike data in 1 ms bins. We then calculated the power spectra for the binned spike and the LFP data, as well as their cross spectra, using multitaper analysis. These spectra and cross-spectra were averaged over trials before calculating coherency. Coherence was obtained by taking the absolute value of the coherency data. All multitaper analyses were performed using the Chronux toolbox (www.chronux.org) under Matlab 7.5 (Mathworks), using a time-bandwidth product of TW = 3 with K = 5 tapers. SFC data were Fisher transformed before subjecting them to population analysis and statistical tests. Spike-field phase relationships were also obtained using the multitaper approach. Each frequency of the spectral analysis is associated with a specific phase relationship that dominated this particular frequency. To calculate spike-field phase relationships for the gamma range, we used the phases associated with 36–40, 40–44, and 44–48 Hz, to analyze the spike-field phase relationship for different stimuli and attention conditions.

In addition to the raw stimulus related LFP, we obtained the single-trial baseline spectra over the time period 300–0 ms before stimulus onset for each recording site and the attend-RF versus attend-away condition. From these single-trial spectra the mean baseline power spectrum (*BPS_M_*) and the standard deviation of the baseline power spectrum (*BPS_SD_*) averaged over all trials (i.e., not separated according to where the animal attended to) was calculated. The stimulus-induced (*P_z_*) power spectrum was then calculated as follows:

$$P_{z}=\frac{{\mathrm{PS}}_{M}-{\mathrm{BPS}}_{M}}{{\mathrm{BPS}}_{\mathrm{SD}}}$$

These were obtained for each stimulus and attentional condition. It provides a measure of spectral power that is induced by the stimulus.

Additionally, we calculated the stimulus-induced spectral power by subtracting, on a trial-by-trial basis, the spontaneous spectral power from the stimulus-induced spectral power. We then calculated the mean spectral power from these “normalized” individual trial spectral powers and divided this by the standard deviation of the spontaneous power obtained from those trials that were available to calculate the stimulus power (i.e., the standard deviation of the spontaneous power was calculated separately for the two attention conditions and each bar length/contrast). The latter approach was done to eliminate possible “random” fluctuations in the LFP baseline signal across individual trials, because each condition is normalized by its corresponding baseline. All quantitative data presented in the paper are based on the first approach, but both approaches yielded virtually identical data.

## Figures and Tables

**Table 1:** Analysis of the Effect of Attention on Different Frequency Bands for the Different Monkeys and Experimental Conditions

Experiment	Animal	Variable	Frequency Band		
			Alpha	Beta	Gamma
Contrast	Monkey HU (n = 39)	raw power	p < 0.001	p < 0.001	p = 0.010
		stimulus power	p = 0.002	p = 0.004	p = 0.004
		STA power			p < 0.001
		SFC			p = 0.023
	Monkey HO (n = 26)	raw power	p = 0.167	p = 0.342	p = 0.026
		stimulus power	p = 0.048	p = 0.82	p = 0.002
		STA power			p < 0.001
		SFC			p = 0.009

Bar length	Monkey BL (n = 12)	raw power	p < 0.001	p < 0.001	p = 0.002
		stimulus power	p < 0.001	p < 0.001	p = 0.005
		STA power			p = 0.013
		SFC			p = 0.011
	Monkey HU (n = 135)	raw power	p < 0.001	p < 0.001	p < 0.001
		stimulus power	p < 0.001	p < 0.001	p < 0.001
		STA power			p < 0.001
		SFC			p = 0.027^∗^
	Monkey HO (n = 46)	raw power	*p < 0.001*	*p < 0.001*	p = 0.518
		stimulus power	*p* = *0.003*	*p < 0.001*	p = 0.265
		STA power			p = 0.006
		SFC			p = 0.102

p values printed in Roman indicate that attention significantly reduced the spectral power and spike field coherence (SFC) in the respective frequency band. p values printed in italics (only monkey HO in the bar length experiment) indicate that attention *increased* the power in the relevant frequency band. An asterisk (^∗^) denotes that attention itself did not have a significant effect on the variable of interest, but that there was a significant interaction between attention and the stimulus (bar length in that instance). The table shows that attention generally reduced the gamma LFP power and SFC in the gamma range. An exception are the data from monkey HO in the bar length experiment, although even here the spike triggered average (STA) LFP power in the gamma range was significantly reduced with attention. The differences between this and the other monkeys for the gamma frequency range may be due to different behavioral strategies. Evidence for this is provided in Figure S2. Only 12 experiments are available from monkey BL due to the fact that the animal's implant had to be removed midway through the experiments.

## Supplemental Information

Document S1. Supplemental Figures and Experimental Procedures
