# Supplementary material for: Attention Reduces Stimulus-Driven Gamma Frequency Oscillations and Spike Field Coherence in V1
Source: Neuron. 2010 Apr 15;66(1-2):114–25. doi: 10.1016/j.neuron.2010.03.013 (PMC2923752; doi:10.1016/j.neuron.2010.03.013)
Supplement: Document S1. Supplemental Figures and Experimental Procedures [file mmc1.pdf]

Neuron, volume *66*

## Supplemental Information

# Attention Reduces Stimulus-Driven Gamma Frequency Oscillations and Spike Field Coherence in V1

**Matthew Chalk, Jose L. Herrero, Mark A. Gieselmann, Louise S. Delicato, Sascha Gotthardt, and Alexander Thiele**

## *Supplemental Experimental Procedures*

**Additional stimulus details**. (1) Bar length experiment: In monkey HO the contrast of the bar was 85% relative to the background, while in monkeys HU and BL it was 24%. The contrast of the patch that the animals needed to detect was 47% relative to the background and 95% relative to the bar in monkey HO, while it was 13% relative to the background and 35% relative to the bar in monkeys HU and BL respectively. These differences may have resulted in different strategies in monkey HO vs. monkey HU and BL in the bar length experiment (see supplementary data). (2) Contrast experiment: In the contrast experiment the contrast of the patch relative to the background was fixed at 7% for both monkeys. The contrast relative to the bar varied with bar contrast. The size of the bar differed between monkeys in the contrast experiment. It was 0.1*0.4 deg large for monkey HU, while it was Monkey HO it was 0.1*1.2 deg.

## *Supplemental Figures*

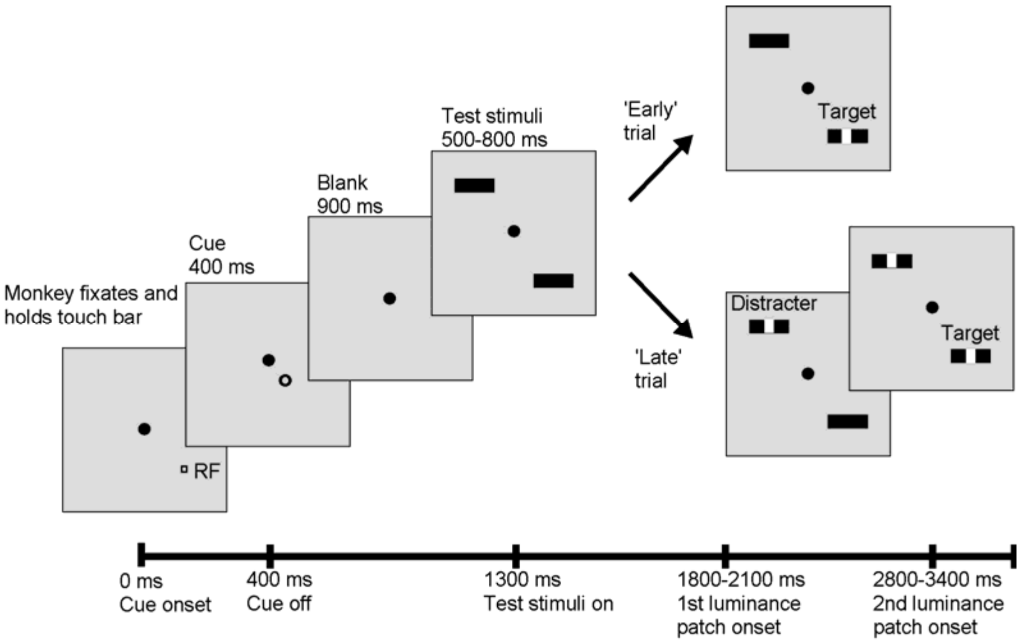


**Supplementary figure 1**: Behavioral paradigm: Monkeys had to fixate and hold a touch bar. Thereupon a cue appeared which indicated where to attend (in the example shown the animal would have to attend to the stimulus within the receptive field). Following a blank period two stimuli were presented, one in the receptive field of the neuron under study another in the opposite hemifield. The animal had to detect a luminance change in the cued location and ignore luminance changes in the un-cued location. The luminance change always occurred at the centre of the bars, i.e. there was only temporal and no spatial uncertainty regarding the valid change.

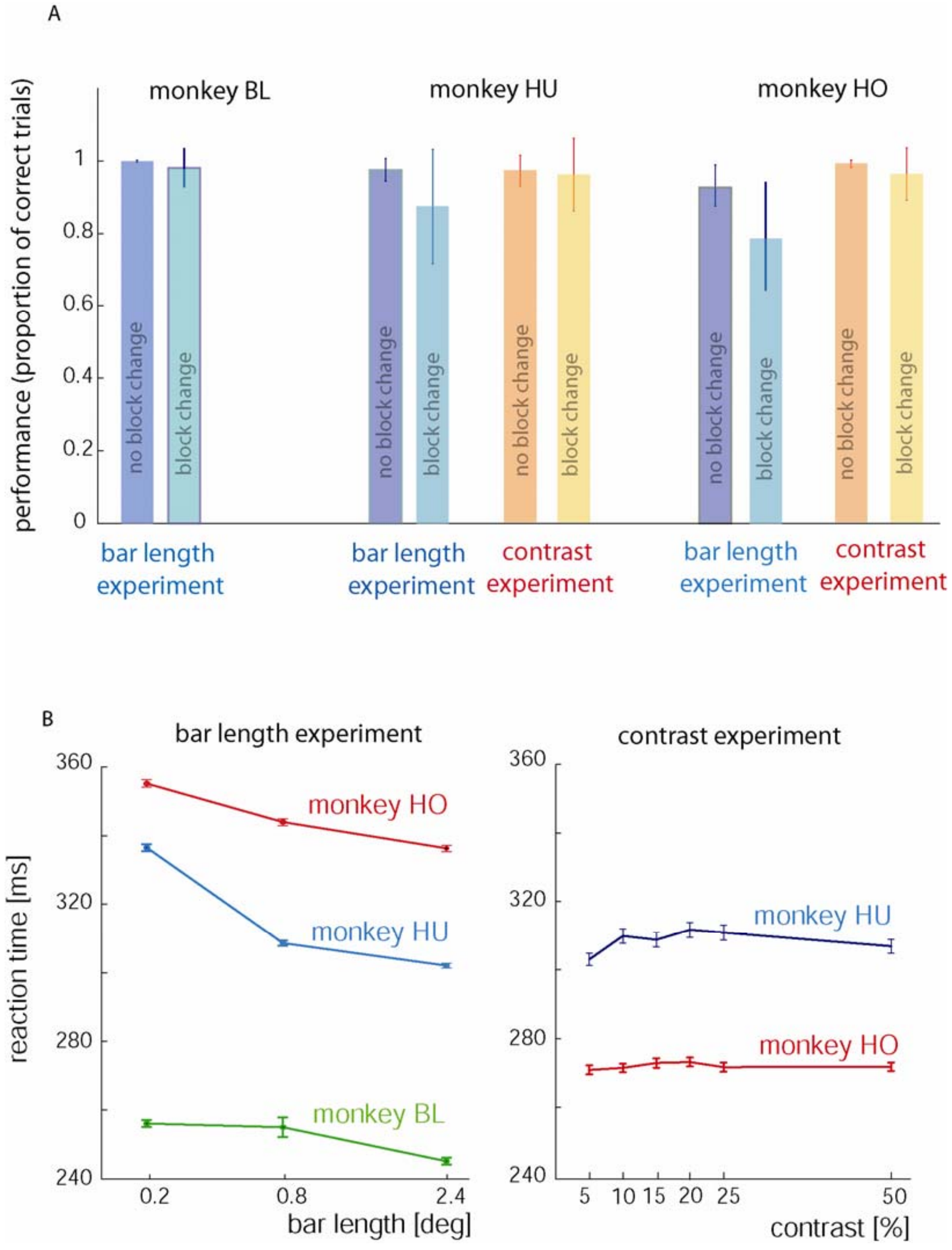


**Supplementary figure 2, related to table 1**: Mean performance and reaction time as a function of experimental task (bar length experiment and contrast experiment). A) Animals' performance in the different tasks. Within block ('no block change') performance indicates the performance (proportion of correct decisions excluding fixation errors) when the cued location on the current trial was the same as the cued location on the previous trial (i.e. attention did not need to be shifted). The performance after a block change is indicated by the 'block change' insets (i.e. attention needed to be shifted to the opposite hemifield compared to the last trial). If the animals did not heed the cue, but switched the location of attention when they were not rewarded for their choice (win-stay/ lose-switch strategy) they should have performed close to 0% correct on 'block change' trials. This was not the case for any of the monkeys. All performed >78% correct on block change trials, indicating that they heeded the

cue well. The extent of this differed between monkeys and the specific experiment performed. Monkey B performed almost at the same rate on cue-stay as on cue (block) change trials (RM-ANOVA, p=0.949) at almost perfect performance. He performed at 99.9% correct on trials where the cue location did not change. On cue change trials the animal performed at 98% correct. Monkey HU's performance on trials where attention did not need to be switched (no block change trials) was 97.5% correct (bar length experiment). On block change trials monkey HU still performed at 87.4% correct. Monkey HO also performed very well, but significantly worse than monkey BL and HU in the bar length experiment. On trials where the block (cue location) did not change, he performed at 92.7 % correct, while on block change trials he performed at 78.6% correct. Its performance was significantly worse compared to monkey BL's and monkey HU's performance (p<0.001, one way ANOVA on ranks). Importantly, monkey HO performed much better in the contrast experiment, where the effects of attention on LFP gamma power and spike field coherence were identical to monkey HU and reflected the pattern seen in the bar length experiment in monkeys BL and HU (see table 1, main text). In the contrast experiment monkey HO performed at 98.9% correct on trials where the block (cue) did not switch and he performed at 96.0% correct on cue change trials. This was very similar to monkey HU's performance in the contrast experiment (97.0% correct on trials where the cue did not switch, 95.9% correct decisions on trials where the cued location changed). In the contrast experiment Monkey HO's performance was actually significantly better than monkey HU's performance on trials where the cue did not change (p=0.007, rank sum), while the performance on cue change trials did not differ significantly (p>0.05, rank sum test).

B) Evidence to support the idea that monkey HO may have deployed less attention to the cued location in the bar length experiment also comes from reaction time data. Of the three monkeys, monkey BL showed by far the fasted reaction times, despite showing the highest accuracy of all three animals. Monkey HU was still significantly faster than monkey HO (p<0.001, rank sum test), despite showing better performance in terms of accuracy. Thus neither monkey BL nor monkey HU demonstrated a speed-accuracy trade off in the bar length experiment. Quite the opposite, the animal that performed the best, and likely paid the most attention, also exhibited the fasted reaction times.

In the contrast experiment the reaction times for monkey HO were dramatically faster compared to the bar length experiment, despite the fact that the patch appearance was harder to detect (it had a lower contrast relative to the background and relative to the bar), and despite the fact that the animal's overall performance had increased. The reaction times for monkey HO in the contrast experiment were significantly faster than monkey HU's reaction times (p<0.001, ANOVA). From these behavioural data it is tempting to speculate that the absence of significant effects of attention on gamma power LFP and spike field coherence in monkey HO in the bar length experiment (see table 1 main text) was a result the animal's strategy to solve the task. It may not have deployed full attentional resources to the cued location in the bar length experiment Conversely, in the contrast experiment, where the animal's performance was significantly better and faster, probably due to attending more strongly to the cued location, the animal showed significant down-modulation of LFP gamma power and spike field coherence when attending to receptive field of the recorded neurons, in line with the results from the other animals.
